# Surveillance Compliance and Quality of Life Assessment Among Surgical Patients with Familial Adenomatous Polyposis Syndrome

**DOI:** 10.1007/s44197-023-00171-8

**Published:** 2024-01-03

**Authors:** Noura Alhassan, Hadeel Helmi, Abdullah Alzamil, Afraj Alshammari, Atheer Altamimi, Sulaiman Alshammari, Thamer Bin Traiki, Saleh Albanyan, Khayal AlKhayal, Ahmad Zubaidi, Omar Al-Obeed

**Affiliations:** 1 Department of Surgery, College of Medicine, King Saud University, Riyadh, Saudi Arabia; 2 College of Medicine, King Saud University, Riyadh, Saudi Arabia; 3 College of Medicine, Princess Nourah Bint Abdulrahman University, Riyadh, Saudi Arabia; 4 Department of Internal Medicine, College of Medicine, King Saud University, Riyadh, Saudi Arabia

**Keywords:** Familial adenomatous polyposis syndrome, Hereditary colorectal cancer, Surveillance, Compliance, Quality of life

## Abstract

**Background:**

Familial adenomatous polyposis (FAP) syndrome has a near-100% lifetime risk of colorectal cancer. Early surveillance and prophylactic surgery have been advocated to reduce this risk. However, the surveillance practices among FAP individuals in Saudi Arabia are unknown. We aimed to explore surveillance compliance in our population, as well as the disease impact on their quality of life (QoL).

**Methods:**

All patients with FAP who underwent surgical resection at King Saud University Medical City between 2016 and 2022 were included. Demographic data, clinical features, family history, and compliance with surveillance were collected and analyzed. QoL questionnaires: Short-form health survey (SF-36) and European Organization for Research and Treatment (EORTC) were conducted by phone interview.

**Results:**

A total of 14 patients were included with an average age of 25 years. Three patients (21.4%) were the first of their family members to develop FAP. Nine patients (64%) were untested for genetic mutation due to lack of referral to geneticists. The compliance rate toward both pre-operative colonoscopy and upper endoscopy were 78%. However, 38% and 27% compliance rates were observed toward initial and post-operative colonoscopy, respectively. The compliance rate was 14% toward thyroid ultrasound. QoL scores varied among patients, with a mean score above 60 across all SF-36 domains.

**Conclusion:**

An overall poor compliance was observed among our participants, particularly toward thyroid ultrasound. Increased health awareness and patient education are essential. In addition, the importance of surveillance and genetic counseling should be emphasized among physicians treating these patients.

## Introduction:

Familial adenomatous polyposis (FAP) is the second most common hereditary colorectal cancer syndrome [1]. It is among one of the few cancer susceptibility diseases that has a near-100% lifetime risk of penetration unless prophylactic surgery is performed [2]. Classical FAP and its variants are caused by mutations in the adenomatous polyposis coli (APC) gene. The condition is inherited in an autosomal dominant manner and predisposes individuals to develop hundreds to thousands of colonic adenomatous polyps as well as early development of colorectal cancer (CRC) compared to the general population [3, 4]. In affected individuals, the average age of polyps’ onset is 16 years, while the average age of CRC diagnosis is 39 years [5, 6]. Although FAP can be diagnosed clinically, genetic confirmation and screening of at-risk relatives are offered. The benefits of genetic testing include the avoidance of the expense, burden, and risk of repeated colonoscopies if the APC mutation is not present [5, 6]. In addition, genetic confirmation aids in ruling out other polyposis syndromes that may exhibit a similar clinical picture.

Due to the high penetrance of CRC, the national comprehensive cancer network (NCCN) recommends surveillance of FAP individuals and at-risk relatives by high-quality colonoscopy in its guidelines. This has to be performed annually starting at the age of 10–15 years [2]. Prophylactic colectomy is usually recommended between the ages of 15 and 25 years, despite recent trends toward individualized surgical timing based on symptomatology and disease burden [7]. Surgical options include total colectomy with ileorectal anastomosis (IRA) or total proctocolectomy (TPC) with ileal pouch-anal anastomosis (IPAA), each is associated with a set of advantages and disadvantages. Although TPC with IPAA is associated with lower risks of acquiring rectal cancer, it is considered a more complex procedure with higher incidence of bladder and sexual dysfunction. On the other hand, IRA provides a lower risk of urgency and fecal incontinence, and preserves fertility in young women because rectal dissection is avoided. However, it is linked to a higher chance of developing rectal cancer, thus requiring more vigilent surveillance post-operatively [2]. Other, less commonly utilized options include TPC with end ileostomy. Continued post-colectomy surveillance is recommended by lower endoscopy every 6–12 months unless a TPC with end ileostomy is performed [2].

The most common extra-colonic manifestation among FAP individuals is upper gastrointestinal polyps, in approximately 50% of patients [7]. Gastroduodenal surveillance with baseline esophagogastroduodenoscopy (EGD) is initiated at 20–25 years of age and follow-up intervals are based on initial findings [2]. Other less common extra-colonic manifestations such as thyroid cancer, osteomas, desmoid tumors, hepatobiliary and central nervous system (CNS) tumors are reported in approximately 70% of FAP patients [8]. NCCN recommendations include baseline thyroid ultrasound (US) in late teenage years, which is to be repeated every 2–5 years if normal. Patients should be considered for abdominal imaging with contrast annually if there is a history of symptomatic desmoid tumor. With regards to CNS tumors, patients should be educated regarding the signs and symptoms of neurological cancer and report to their physician if they develop any [2].

Despite the usefulness of surveillance in lowering the risk of future cancer in FAP patients, the literature shows that compliance of FAP individuals has been variable [9, 10]. Reasons behind non-compliance may include poor health education, lack of health insurance for financial coverage, or discomfort related to colonoscopy. Another prominent reason behind non-compliance to surveillance in multiple reports is the participants’ perception toward their cancer risk despite the known fact of 100% penetrance of CRC among FAP [9, 10]. Furthermore, FAP is predicted to have an impact on quality of life (QoL) and daily activities such as work, recreational activities, and relationships [10]. The aim of this study was to assess the compliance rate of FAP patients who underwent surgical resection toward the recommended surveillance modalities and to evaluate their QoL.

## Methodology

### Study Design and Sample

This case series was conducted to assess the compliance and QoL among FAP/attenuated FAP patients. Patients who underwent colorectal surgery at King Khalid University Hospital, King Saud University Medical City in Riyadh, Saudi Arabia were screened. Patients who underwent surgery for FAP between June 2016 and June 2022 were identified and included.

### Data Collection Methods

Patient demographic data including age, gender, body mass index (BMI), and medical comorbidities were extracted from patient medical records. In addition, past surgical history and results of previous surveillance investigations were recorded.

A telephone interview was conducted by the data collectors to collect further details regarding patients’ level of education, occupation, and income. A detailed personal and family history of FAP and /or CRC were obtained from each participant. The participants were questioned regarding their surveillance attitudes; patients were asked if they had ever been advised for genetic testing or surveillance by a doctor or other healthcare professional. Finally, QoL was assessed using two validated questionnaires: the Short-form health survey (SF-36) and the European Organization for Research and Treatment (EORTC) quality of life questionnaires (EORTC QLQ-CR29) [11, 12]. Both questionnaires existed in Arabic forms and did not require translation. Permission was granted prior to utilizing both questionnaires.

### Definitions

Participants’ compliance was evaluated using the following methods based on NCCN guidelines [2]. Colorectal compliance was divided into three types based on the patient's condition. Initial colorectal surveillance was assessed in all patients with a prior family history of FAP and defined as having their first colonoscopy between the ages of 10–15 years. Patients with no prior family history of FAP were excluded from this assessment. Second, pre-operative colorectal surveillance was assessed by calculating the number of colonoscopies performed between the age at diagnosis and the age at surgery. An adequate number was considered when a patient had a colonoscopy at least annually between the time of diagnosis and the time of surgery. Finally, post-operative compliance was measured as having a colonoscopy or proctoscopy within the last year for the patients who had undergone IRA or IPAA. Those who underwent TPC with end ileostomy were excluded from this measure.

Extra-colonic compliance was measured toward gastroduodenal polyps/cancer and thyroid cancer. Based on the Spigelman classification, patients were considered compliant if they were evaluated by EGD within 1–4 years based on their findings [13]. Patients were considered compliant if they had a thyroid US within the last 2–5 years.

### Statistical Analysis

Data were analyzed using Statistical Package for Social Studies (SPSS 22; IBM Corp., New York, NY, USA). Continuous variables were expressed as mean ± standard deviation and categorical variables were expressed as percentages. Mann–Whitney Test and Kruskal–Wallis Test were used. A *p*-value < 0.05 was considered statistically significant.

### Ethical Consideration

The institutional review board approval for this study was obtained before collecting the data. Verbal consent was obtained from study participants, and their privacy was respected throughout the study.

## Results

A total of 14 patients with FAP who underwent surgical resection were included in the current analysis. Demographic data and family history are shown in Table 1. The majority of the patients (64%) were females with a mean age of 32 years at the time of surgery. Approximately 21% of patients were the first member of their families to be diagnosed with FAP. A family history of CRC was positive in 12 patients (85.7%).

**Table 1:** Demographic characteristics and family history

Variables	Number	%
Age (Mean, SD)	34.93	12.83
BMI (Mean, SD)	26.46	5.51
Gender		
Male	5	35.7
Household income		
< 5000 SAR	6	42.9
5000–15000 SAR	6	42.9
> 25,000 SAR	2	14.3
Highest level of education		
High school	9	64.3
Diploma	2	14.3
Bachelor’s degree	3	21.4
Occupation		
Full-time employee	6	42.9
Student	2	14.3
Retired	3	21.4
Unemployed	3	21.4
Medical comorbidities		
Bronchial asthma	6	42.9
Hypertension	1	7.1
Hyperthyroidism	1	7.1
Family history of polyps	11	78.6
Family history of FAP	11	78.6
Number of first-degree relatives with FAP		
2	4	28.6
3	3	21.4
> 3	4	28.5
Number of second-degree relatives with FAP		
0–10	8	57.1
10–20	2	14.3
> 20	1	7.1
Family history of CRC	12	85.7

*SD* standard deviation, *BMI* body mass index, *SAR* Saudi Arabian Riyals, *FAP* familial adenomatous polyposis, *CRC* colorectal cancer

Table 2 presents FAP-related clinical features and surgical interventions. Almost all patients (93%) had classical FAP, while 1 was diagnosed with attenuated FAP. The mean age at diagnosis was 25 ± 12 years. More than half (64%) of the patients were untested for the APC mutation. Three patients (21.4%) had a diagnosis of CRC; out of whom, 2 patients were the first of their family to develop FAP. They were diagnosed with FAP at the ages of 33 and 47 years, respectively, and were initiated on a surveillance plan since then. Although they were compliant to pre-operative colorectal surveillance, CRC was detected during surveillance. The third patient was found to have cancer on final pathology following prophylactic surgery. Gastroduodenal polyps were identified in 57% of the patients. Most patients (79%) had a colonoscopy as the first modality of surveillance, while the remaining had initial surveillance with sigmoidoscopy. The mean age at first scope was 28 ± 10 years, with 5 ± 2 number of surveillance scopes done for each patient on average. The mean age at surgery was 32 ± 12 years. Eight patients (57%) underwent TPC with IPAA, 5 (36%) underwent total colectomy with IRA and 1 (7%) patient had a TPC with end ileostomy, with 64% of patients having underwent rectal dissection. Seven patients (50%) developed post-operative complications. The most common complication was post-operative ileus reported among 4 patients (28.6%) followed by bowel obstruction in 2 patients (14.3%). Three patients (21%) required re-admission for either re-intervention or re-operation.

**Table 2:** Clinical features and surgical interventions

Variables	Number	%
Age at diagnosis (Mean, SD)	25.43	11.58
Age at first scope (Mean, SD)	27.57	10.52
Age at surgery (Mean, SD)	32.29	11.72
APC mutation status		
Confirmed by genetic testing	5	35.71
Presence of extra-colonic manifestations	8	57.14
Gastroduodenal polyps	8	57.14
Site of GD polyp		
Gastric	2	14.29
Duodenal	5	35.71
Both	2	14.29
Type of GD polyp		
Adenomatous	3	21.43
Hyperplastic	1	7.14
Both	2	14.29
Desmoid tumor	1	7.14
Colorectal cancer	3	21.43
Type of surgery		
TPC with IPAA	8	57.1
Total colectomy with IRA	5	35.7
TPC with end ileostomy	1	7.1
Rectal dissection	9	64.3

*SD* standard deviation, *APC* adenomatous polyposis coli, *GD* gastroduodenal; *TPC* total proctocolectomy, *IPAA* ileal pouch-anal anastomosis, *IRA* ileorectal anastomosis

The compliance toward different forms of surveillance is shown in Table 3. The highest level of compliance was observed toward pre-operative colorectal surveillance as well as EGD surveillance (*n* = 11, 78%). The lowest compliance was for thyroid disease surveillance that was conducted using thyroid ultrasound in 2 patients (14%).

**Table 3:** Compliance among patients

Variable	Definition	Number	%
Initial compliance*	First colonoscopy at the ages of 10–15 years	3/11	27.27
Pre-operative compliance	Annual colonoscopy between diagnosis and surgery	11/14	78.57
Post-operative compliance**	Colonoscopy within 1 year	5/13	38.46
EGD compliance	EGD within 1–4 years based on Spigelman classification	11/14	78.57
Thyroid compliance	Thyroid US within 2–5 years	2/14	14.29

*EGD* Esophagogastroduodenoscopy, *US* ultrasound

*Patients without prior family history of FAP were excluded

**One patient who underwent total proctocolectomy with end ileostomy was excluded

**Fig. 1:**
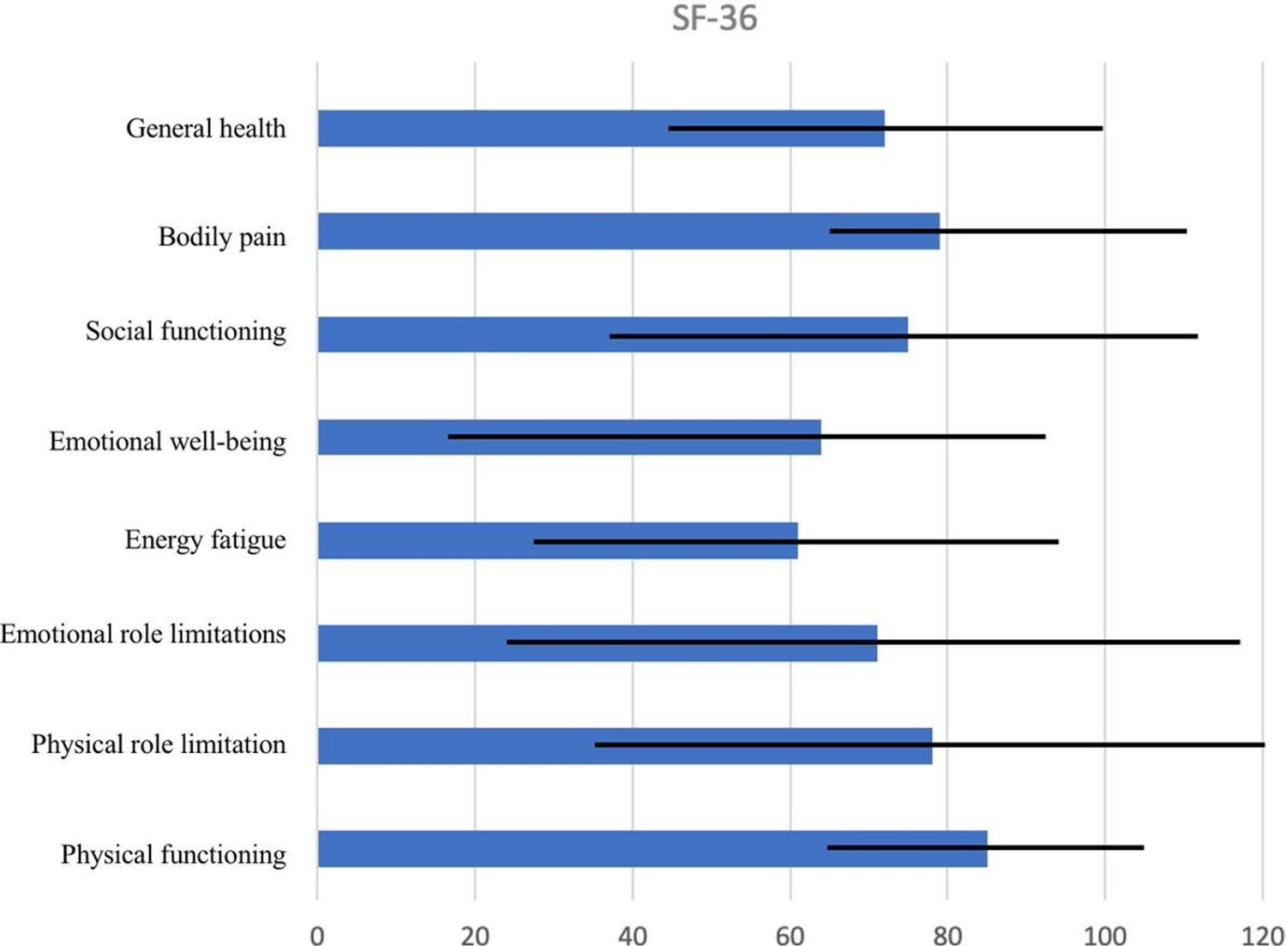
Results of SF-36 QoL among our 14 patients. Higher scores of SF-36 indicate better health status

**Fig. 2:**
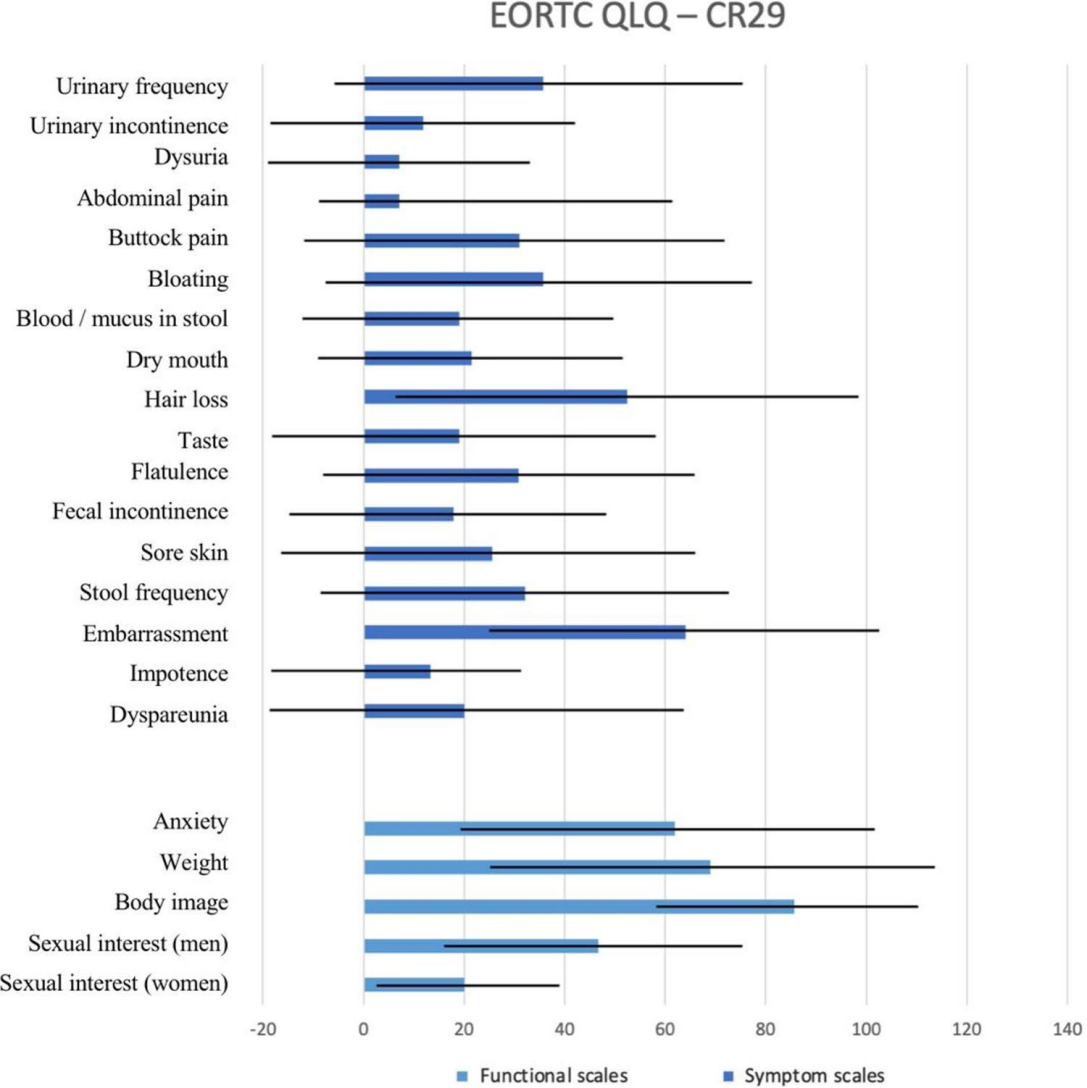
Mean and SD of EORTC QLQ-CR29. Symptom scales: a high score for the symptom scales represents a high level of symptomatology or problems. Functional scales: a high score for the functional scale represents a high level of functioning

QoL scores varied among patients, with overall good scores across all domains on the SF-36 survey. Good scores were also reported for urinary and gastrointestinal symptoms on the EORTC QLQ – CR29 scale. The highest level of symptomatology reported was embarrassment, followed by hair loss. The single patient with a stoma did not report any stoma-related problems. Figures 1 and 2 show further details regarding QoL scores.

## Discussion

FAP is considered the second most common hereditary CRC syndrome, with a 100% lifetime chance of developing CRC unless prophylactic surgery is performed [1, 2]. There is a substantial deficiency in the reports from Saudi Arabia on this topic with lack of research on the prevalence, clinical aspects, genetic factors, and management of FAP. This study was designed to evaluate the adherence of FAP patients to the recommended surveillance, based on NCCN guidelines [2]. Poor overall compliance was observed, particularly toward thyroid ultrasound. The highest compliance was found toward pre-operative colonoscopy and upper endoscopy. The compliance rates to initial and post-operative colorectal surveillance were 38% and 27%, respectively. Additionally, an assessment of QoL among these participants showed overall good scores, particularly on the SF-36 scale.

Although the diagnosis of FAP can be suggested based on clinical findings, genetic confirmation is advocated in suspected individuals as well as screening of all first-degree relatives [2]. The advantages of genetic testing include the avoidance of the cost, inconvenience, and risks associated with repeated colonoscopies in individuals who prove to be unaffected [5, 6]. Furthermore, lack of genetic confirmation has been linked to non-compliance to surveillance colonoscopy [9]. Throughout the literature, previous studies have reported rates of genetic testing among FAP individuals and at-risk relatives that were ranging between 40 and 60% [9, 14, 15]. Among our study participants, 36% tested for the APC mutation. Lack of referral to geneticists by the primary treating physicians may play a role. In addition, the public still has reservations toward genetic testing despite the availability of well-established genetic counseling centers and advanced testing modalities [17, 18]. Fear of stigmatization among affected families is a commonly encountered challenge faced by genetic counselors [18].

The compliance of FAP individuals toward recommended surveillance is poorly documented in the literature with most studies focusing on compliance toward colonoscopy. The compliance rates toward surveillance colonoscopies range from 54 to 84% in the literature [9, 10, 15]. The favorable compliance rates among certain studies could be attributed to the presence of a national registry for hereditary cancer as well as a physician-notification system for missed endoscopy appointments [9]. Furthermore, when compared to FAP patients, at-risk relatives were considered more compliant in one study but less compliant in another, with compliance rates of 87.5% and 42%, respectively [9, 15]. Since surveillance initiation is suggested at a young age of 10–15 years, compliance may also be influenced by age. In a study assessing surveillance trends among minors, only 33% of child FAP participants had a previous colonoscopy, even though 51% of their parents had been provided with surveillance recommendations from a healthcare professional [14]. Furthermore, FAP individuals may be reluctant to pursue medical care or follow surveillance recommendations as long as they remain asymptomatic. Poor compliance may be influenced by deficient counseling by the treating healthcare provider who is delivering treatment. In one study, participants who had not received strong clear recommendation by a healthcare professional to perform colonic examination were 4.8 times less likely to have had one [9]. They reported that 94% of FAP participants had received some sort of advice by a healthcare provider to perform colonoscopy, which is comparable to 100% among our individuals [9]. However, the exact quality or source of counseling/advice was not assessed in either study. Post-operative surveillance for FAP patients should include an evaluation by lower endoscopy every 6–12 months for patients undergoing IRA or IPAA [2]. We observed lower compliance with post-operative surveillance compared to the literature with only 38.46% of participants having had formal lower endoscopic evaluation within the past year. In a previous report, only 14% of participants did not follow post-operative endoscopic surveillance recommendations [19]. Another study limited to patients with prior IRA showed that 74% of individuals had a recent endoscopic evaluation [10]. The low compliance observed among our participants may be explained by the use of bedside methods of evaluation such as rigid sigmoidoscopy or anoscopy that would have been missed in our assessment.

Peri-ampullary cancer is one of the most common causes of death in those who have underwent prophylactic colectomy for FAP [20]. In addition to pre-operative colorectal surveillance, EGD surveillance had the highest compliance among our population reaching 78.57%. Only one of the previously mentioned studies shed light on EGD compliance, with 80% of their FAP participants reported having an upper endoscopy at any point during their lifetime [15]. More than half of our patients had gastroduodenal polyps (57%) which may have played a role in our observed good compliance. Additionally, at our institution sigmoidoscopies are performed in the endoscopy unit, which eases the scheduling of both upper and lower scopes at once.

Although the risk of thyroid malignancy is low, 1–2% of thyroid cancer cases have been linked to FAP, and surveillance for this type of cancer is part of the NCCN recommendations [2, 21]. To our knowledge, no FAP studies in the literature have included compliance toward thyroid disease. In fact, we have observed that compliance toward this was the lowest with only two patients (14%) undergoing thyroid US within 5 years of the study. This could be explained by the low incidence of the disease.

FAP, similar to other hereditary cancer syndromes, is predicted to affect an individual’s QoL and daily living whether socially or personally. Emotions of hopelessness have been observed among FAP patients, particularly individuals with lower QoL scores [22]. Although the QoL of individuals with or at-risk for FAP was comparable to the general population in some studies [10]. FAP has been linked to limitations in social interactions, job opportunities, and daily activities in previous studies [23–25]. Among our participants, we observed good QoL scores particularly on the SF-36 scale, with the highest score in physical functioning (mean = 85). This is consistent with the results noted on the EORTC QLQ-CR29 scale, where urinary and gastrointestinal complaints were low, even though the majority of patients had a history of surgery with rectal dissection. All the surgeries were performed laparoscopically which may have favorably affected this. The highest symptomatology among the EORTC QLQ-CR29 scale was reported toward embarrassment, which does not coincide with the previous scale showing good social and emotional well-being.

Prophylactic surgery reduces the cancer risk among FAP individuals, and may also alleviate the symptom burden in certain patients. In one study, patients with FAP who underwent surgery had significantly lower QoL scores on the SF-36 scale compared to those who have not had surgery, and noncarriers. Using the EORTC QLQ-CR29 scale, they also found that defecation problems and body image were significantly affected among the post-surgical group, although the type of operation did not affect QoL outcomes [10]. Other studies show mixed reports when comparing QoL among patients who underwent IRA and IPAA, whether for FAP or other conditions [26–28]. Due to the small sample size, we could not find any correlations between QoL scores and type of surgery. Among post-surgical patients, recurrent hospital visits for continued surveillance are of concern whether due to the distress of the surveillance procedure or because the hospital visit served as a reminder of their potential for further deterioration [25].

Our study highlights the lack of surveillance compliance among FAP individuals despite the availability of standardized clinical guidelines. Enhancing patients’ awareness of their condition and promoting genetic testing and surveillance among family members should be emphasized in the Kingdom. Additionally, spreading awareness among involved healthcare providers, including surgeons, gastroenterologists and family physicians would improve compliance rates. Efforts to establish a national registry should be sought out to ease the diagnosis, genetic counseling and monitoring of FAP individuals and at-risk relatives.

## Limitations

There are several limitations to our study. Our initial study plan was to use our surgical database to identify FAP individuals, then further extend the sample to include all first-degree family members of those individuals. All participants refused to share the contact information of their affected relatives, which reduced the number of recruited subjects and ultimately our sample size. The small sample size limits the generalizability of the results and our ability to detect predictors of compliance or QoL. In addition, only patients diagnosed with FAP and had prior surgery were included, which might have affected the QoL scores. Further research including a larger sample size and non-surgical patients is advised to fully assess our research question.

## Conclusion

Among 14 FAP patients who underwent surgical intervention, the overall compliance was poor across most of the domains. The highest compliance was toward pre-operative colorectal and EGD surveillance. This overall poor compliance was observed despite all patients having received healthcare recommendations for surveillance. Increased awareness about FAP syndrome among the general population as well as healthcare providers is essential. Implementation of mandatory genetic testing among FAP patients and at-risk relatives may help detection and surveillance.

## Data Availability

The data supporting the conclusion of this article will be made available by the corresponding author, upon request.

## Abbreviations

APC: Adenomatous polyposis coli
BMI: Body mass index
CNS: Central nervous system
CRC: Colorectal cancer
EGD: Esophagogastroduodenoscopy
FAP: Familial adenomatous polyposis
IPAA: Ileal pouch-anal anastomosis
IRA: Ileorectal anastomosis
NCCN: National comprehensive cancer network
QoL: Quality of life
TPC: Total proctocolectomy
US: Ultrasound
